# Population tailored modification of tuberculosis specific interferon-gamma release assay

**DOI:** 10.1016/j.jinf.2015.10.012

**Published:** 2016-02

**Authors:** Kata Horvati, Szilvia Bősze, Hannah P. Gideon, Bernadett Bacsa, Tamás G. Szabó, Rene Goliath, Molebogeng X. Rangaka, Ferenc Hudecz, Robert J. Wilkinson, Katalin A. Wilkinson

**Affiliations:** a MTA-ELTE Research Group of Peptide Chemistry, Eötvös L. University, Budapest, Hungary; b Clinical Infectious Disease Research Initiative, Institute of Infectious Disease and Molecular Medicine, University of Cape Town, Cape Town, South Africa; c Department of Genetics, Cell- and Immunobiology, Semmelweis University, Budapest, Hungary; d Department of Laboratory Medicine, Semmelweis University, Budapest, Hungary; e Department of Organic Chemistry, Eötvös L. University, Budapest, Hungary; f The Francis Crick Institute Mill Hill Laboratory, London NW7 1AA, UK; g Department of Medicine, Imperial College London W2 1PG, UK

**Keywords:** Tuberculosis, Interferon-Gamma Release Assays (IGRA), QuantiFERON assay, Peptide epitope, Rv2654c, HIV

## Abstract

**Objectives:**

Blood-based Interferon-Gamma Release Assays (IGRA) identify *Mycobacterium tuberculosis* (MTB) sensitisation with increased specificity, but sensitivity remains impaired in human immunodeficiency virus (HIV) infected persons. The QuantiFERON-TB Gold In-Tube test contains peptide 38–55 of Rv2654c, based on data indicating differential recognition between tuberculosis patients and BCG vaccinated controls in Europe. We aimed to fine map the T cell response to Rv2654c with the view of improving sensitivity.

**Methods:**

Interferon-gamma ELISpot assay was used in HIV uninfected persons with latent and active tuberculosis to map peptide epitopes of Rv2654c. A modified IGRA was tested in two further groups of 55 HIV uninfected and 44 HIV infected persons, recruited in South Africa.

**Results:**

The most prominently recognised peptide was between amino acids 51–65. Using p51-65 to boost the QuantiFERON-TB Gold In-Tube assay, the quantitative performance of the modified IGRA increased from 1.83 IU/ml (IQR 0.30–7.35) to 2.83 (IQR 0.28–12.2; *p* = 0.002) in the HIV uninfected group. In the HIV infected cohort the percentage of positive responders increased from 57% to 64% but only after 3 months of ART (*p* = ns).

**Conclusions:**

Our data shows the potential to population tailor detection of MTB sensitization using specific synthetic peptides and interferon-gamma release *in vitro*.

## Highlights

• Refined epitope mapping of Rv2654c was performed in a South African Xhosa population.
• The most frequently recognized peptide is p51-65 (VRAVAESHGVAAVLF).
• Using p51-65 in the QFT assay resulted in a significant boosting effect.
• Peptide p51-65 can improve the population tailored detection of MTB sensitization.

## Introduction

Despite the worldwide availability of vaccination, diagnosis and treatment, tuberculosis (TB) is still a major global health problem and it is estimated that more than one-third of the world's population is infected by *Mycobacterium tuberculosis* (MTB).^1^ Exposure to MTB may result in latent TB infection (LTBI) which is defined by the absence of clinical TB symptoms but carries about 5–10% lifetime risk of developing active TB and comprises a significant reservoir of future cases of active disease, particularly in countries with high HIV burdens.^2^ The estimated incidence of TB is 9 million each year, among which 1.5 million cases die. HIV infected patients are about 30 times more likely to develop active TB and HIV-TB co-infection is responsible for one fifth of all TB related deaths.

The currently used vaccine – *Mycobacterium bovis* Bacille Calmette Guérin (BCG) – does not protect adults against TB.^3^ Moreover, BCG vaccination renders the TB diagnosis more difficult as the commonly used tuberculin skin test (TST), based on the administration of purified protein derivative (PPD), may give false-positive results due to previous BCG immunization.^4^

Proteins encoded at loci missing from BCG^5^, ^6^ have become of interest for TB diagnosis. Such regions of difference (RD), by definition, are absent from all BCG vaccine strains.^7^ Comparative genomic studies have identified 11 regions of difference of which RD1 contains important immunodominant proteins such as ESAT-6 (Rv3875) and CFP-10 (Rv3874).^8^ These proteins showed reliable diagnostic potential in T-cell based IFN-γ release assays (IGRAs) and are used as antigens in FDA approved and commercially available blood tests such as QuantiFERON-TB Gold In-Tube (QFT) and T-SPOT.TB.^9^ ESAT-6 and CFP-10 are early secreted proteins which form a heterodimeric complex^10^ and contribute to the virulence of MTB.^11^ However, genes for ESAT-6 and CFP-10 were found not only in MTB but also in virulent *M. bovis*, and in several non-tuberculous mycobacteria.^12^, ^13^ Thus, infection with such ESAT-6 or CFP-10 expressing mycobacterial species can result in a false-positive response in blood tests based on ESAT-6 and CFP-10 antigens.

MTB sensitization was traditionally described with a model of binary distribution between active TB and latent TB. Evidence supports that the interaction between MTB and the host immune system represents a spectrum of immune responses, bacterial load, metabolic activity and stages of infection ranging from sterilizing immunity to TB disease.^14^, ^15^ Such heterogeneity may explain the fact that antigens that are targets of the immune response in latent TB are frequently found as target in active TB and no adequate differentiation between latent and active disease has been revealed.^16^, ^17^

Insufficient sensitivity of IGRA tests could be further enhanced with the use of more antigens derived from different immunodominant proteins of MTB. Previous studies have demonstrated that Rv2654c can improve specific immune-based diagnosis of TB infection especially in the BCG-vaccinated population^18^, ^19^ and one peptide (p38-55) has been included in the commercially available QuantiFERON-TB Gold In-Tube (QFT) test. Rv2654c is an 81 amino acid alanine-rich protein, encoded in the RD11 region, highly specific for MTB and absent from most of the atypical mycobacteria. We performed a systematic epitope mapping of the Rv2654c protein and found that the most immunogenic peptide recognized by MTB sensitized individuals in South Africa is different from that previously described by Aagaard and co-workers.^18^ We describe the evaluation of a modified QuantiFERON-TB Gold In-Tube test in HIV infected and uninfected cohorts.

## Materials and methods

### MHC-binding predictions

The amino acid sequence of Rv2654c protein was downloaded from the TubercuList^20^, ^21^ website (http://tuberculist.epfl.ch/index.html). *In silico* identification of immunogenic regions of the Rv2654c protein was based on predicting the MHC-binding affinity of 15-mer peptides overlapping by 14 amino acid residues, using the MHC II binding prediction tool^22^, ^23^ available at the Immune Epitope Database (www.iedb.org).^24^ Binding affinities were predicted for the 27 representative MHC II alleles recommended by Greenbaum and colleagues, based on studying peptide binding to MHC supertypes^25^ and evaluated based on the consensus percentile ranks. As suggested by Paul and colleagues,^26^ 15-mer peptides were sorted based on the median value of the percentile ranks predicted for each representative allele and the top scoring ∼20% were identified as possible immunodominant epitopes.

### Synthesis and analytical characterization of peptides

Peptides were produced on solid phase with an automated peptide synthesizer (Syro-I, Biotage, Uppsala, Sweden) using standard Fmoc/tBu strategy. Peptides used in this study contained an amide group at the C-terminus for stability purposes. After cleavage, crude peptides were purified by semi-preparative HPLC and analysed by mass spectrometry, analytical HPLC and amino acid analysis (Table 1). Mass spectrometric analyses were performed on a Bruker Esquire 3000 + ion trap mass spectrometer (Bruker, Bremen, Germany) equipped with electrospray ionization (ESI) source. Samples were dissolved in a mixture of acetonitrile/water = 1/1 (v/v) containing 0.1% acetic acid and introduced by a syringe pump with a flow rate of 10 μL/min. The homogeneity of the compounds was checked by analytical HPLC using a laboratory-assembled Knauer HPLC system (Bad Homburg, Germany) using 1 mL/min flow rate at room temperature. The gradient elution system consisted of 0.1% TFA in water (eluent A) and 0.1% TFA in acetonitrile/water = 80/20 (v/v) (eluent B). Amino acid analyses were performed on a Sykam Amino Acid S433H analyzer (Eresing, Germany) equipped with an ion exchange separation column and post-column derivatisation. Prior to analysis samples were hydrolysed with 6 M HCl in sealed and evacuated tubes at 110 °C for 24 h. For post-column derivatisation the ninhydrin-method was used. Peptides were stored dry until reconstitution as 1 mg/mL stock solutions in 0.5% DMSO containing PBS buffer.

### Selection and description of participants

The University of Cape Town’s Faculty of Health Sciences Human Research Ethics Committee approved this study (REC 296/2007, REC 245/2009). Written informed consent was obtained from all participants.

For epitope mapping of Rv2654c, patients with active TB or latent TB infection (LTBI) were recruited at Ubuntu Clinic, site B Khayelitsha, South Africa. All were of Xhosa ethnicity. Active TB was defined by smear and/or culture positivity for MTB from one or more sputum specimens. LTBI was defined by an IFN-γ ELISpot response to ESAT-6 or CFP-10 of 20 spot-forming cells (SFC)/10^6^ PBMC above background, in the absence of clinical symptoms or radiographic abnormality and with negative sputum smear and culture for *M. tuberculosis*. All subjects recruited for epitope mapping underwent HIV counselling and testing, and positivity was an exclusion criterion, together with pregnancy and age under 18 years.

For the evaluation of the boosting effect of peptide 51-65 on the QuantiFERON-TB Gold In-Tube test, 55 HIV uninfected and 50 HIV infected persons were recruited at Ubuntu Clinic, Site B Khayelitsha, South Africa. HIV infected patients were recruited at enrolment into the antiretroviral treatment (ART) programme based on their eligibility (CD4^+^ T cell count of 250 cells/ml or less), according to South African national guidelines at the time. 30 ml venous blood was collected for immunological analysis, as well as viral load and CD4^+^ T cell count measured at day 0, 1 month, 3 months and 6 months of ART. Induced sputum was collected at all time-points; six patients with positive MTB culture were referred to TB treatment and excluded from further follow up and analysis.

### Cell culture and *in vitro* assays

Peripheral blood mononuclear cells (PBMC) were prepared using standard Ficoll separation technique and were stored in liquid nitrogen until used. The measurement of IFN-γ by ELISpot was performed as described previously^27^, ^28^ using the human Interferon-γ ELISpot^PRO^ kit (MABTECH, Nacka Strand, Sweden). Briefly, 2.5 × 10^5^ PBMC were plated in 200 μL of RPMI culture media supplemented with 10% FCS. Antigenic stimuli were either Rv2654c peptides at 10 μg/mL final concentration, or a pool of ESAT-6 (Rv3875, 5 μg/mL) and CFP10 (Rv3874, 5 μg/mL) derived peptides. As positive control, anti-CD3 mAb CD3-2 at 100 ng/mL final concentration was used. ELISpot plates were read on an Immunospot Series 3B Analyzer (Cellular Technology, Cleveland, OH, USA) and plates were retained for visual inspection and confirmation in the case of anomaly. Results are quoted as spot forming cells per million PBMC (SFC/10^6^ PBMC), with the background (unstimulated) values subtracted.

The QuantiFERON-TB Gold In-Tube test was performed in a Cellestis accredited laboratory according to the manufacturer's instructions (QuantiFERON-TB Gold In-Tube, Cellestis Ltd., Carnegie, Australia). The modified (peptide boosted) QuantiFERON-TB Gold In-Tube test (QFTB) was performed in parallel, using a second set of tubes (representing Nil, Antigen, Mitogen), with peptide 51-65 added at 5 μg/ml final concentration (determined based on dose response experiments) in the laboratory. Tubes were incubated overnight and IFN-γ measured as IU/ml, using the commercial assay. The results were interpreted similarly, using the manufacturer's criteria for assay positivity (≥0.35 IU/mL above nil).

### *In silico* HLA-binding study

In an attempt to relate epitope predictions to our laboratory findings, binding affinities were also corrected for the allele frequencies observed in the Xhosa population. Since the most detailed description of HLA types in the Xhosa population^29^ is based on serological testing, while prediction tools are genotype-based, serotype frequencies were interpreted as allele frequencies according to the conversion matrix provided as Supplementary Table 1.

To estimate the portion of the Xhosa population expected to present the given epitope, we used a model, where an individual's antigen presenting cells (APCs) express both maternal and paternal HLA-DR and HLA-DQ genes. The relative number of individuals expressing a given combination of HLA loci was computed using the Hardy–Weinberg equation.^30^ Competition between peptide epitopes was simulated according to two different scenarios, described in the supplementary information in more detail. Based on these simulations, only the best scoring peptides in a particular subpopulation were regarded as ‘*MHC-binder*’ peptides. The sum of all fractions of the population, where the peptide scored as a ‘*binder’*, was regarded as the portion of the whole population that is able to present the peptide epitope. Results were visualized with the help of the Matplotlib Python module.^31^

In addition to the Xhosa population, HLA frequencies described in the Danish population (a source of donors for testing the p38-55 peptide in a previous study^18^) were also included in the analysis. The frequencies described by Lindblom and colleagues^32^ were downloaded from the Allele Frequency Net Database (allelfrequencies.net).^33^ In this case, a more simplified model was used for simulating the population, omitting HLA-DR3/DR4 and HLA-DQ loci.

### Statistical analysis

Data were analysed using GraphPad Prism v6 software (San Diego, CA, USA). For analysis of statistical significance (*p* < 0.05) the Mann–Whitney U test was used for unpaired data and the Wilcoxon matched pairs test was used for paired data. Bonferroni correction was used to correct *p* values for multiple comparisons where applicable.

## Results

### Identification of highly immunogenic regions by MHC-binding predictions

**Figure 1:**
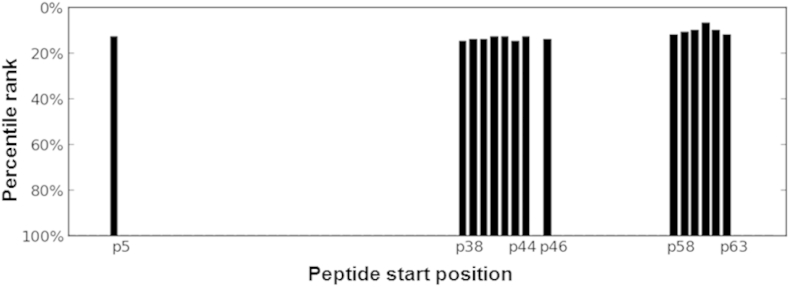
**Identification of highly immunogenic regions by MHC-binding predictions**. The median value of the consensus percentile rank scores of binding to supertype-defining HLA II alleles was obtained from predictions by the IEDB webservice, for each 15-mer peptides.^26^ Since the smaller consensus percentile rank a peptide has, the better binding is expected, percentile rank values were converted to binding scores by subtraction of the value from 100. The 100 minus median rank values are shown for the top scoring 20% (15 peptides) as a function of starting residues.

The median value of the consensus percentile rank scores of binding to supertype-defining HLA II alleles was obtained from predictions by the IEDB webservice, for each 15-mer peptide.^26^ As reflected by the median score for MHC binding, the highest binding peptides of the Rv2654c protein were found to be in the middle section (starting amino acid positions between 36 and 40) and the *C*-terminal section with starting positions between 58 and 63 (Fig. 1).

### Epitope mapping of Rv2654c protein

To validate and refine the *in silico* prediction, synthetic 20-mer peptides, spanning the complete sequence of the Rv2654c protein and overlapping by 10 amino acids, were prepared and analysed *in vitro* (Table 1). A pool of overlapping peptides, covering the sequence of Rv2654c, was used as a substitute for the recombinant protein, as previous studies have demonstrated antigenic equivalence of the T-cell response between the recombinant proteins and the corresponding synthetic peptide mixtures for ESAT-6, CFP-10 and TB10.4.^34^, ^35^

Altogether, PBMC from 34 individuals were used, comprising 24 individuals with LTBI (median age 27 years, 14 female, 10 male) and 10 patients with active TB (median age 27 years, 4 female, 6 male). Since all individuals were MTB sensitized, and there was no differential recognition of the peptides between LTBI and active TB patients in line with published literature,^16^, ^17^ results were combined for analyses (Table 2). The frequency of recognition of the C-terminal peptides p51-70 and p61-81 was comparable to that of the Rv2654c pool in the combined groups (29% and 35% compared to 32% respectively). We therefore selected the *C*-terminal 51–81 region of the protein for further characterization, using shorter peptides.

**Figure 2:**
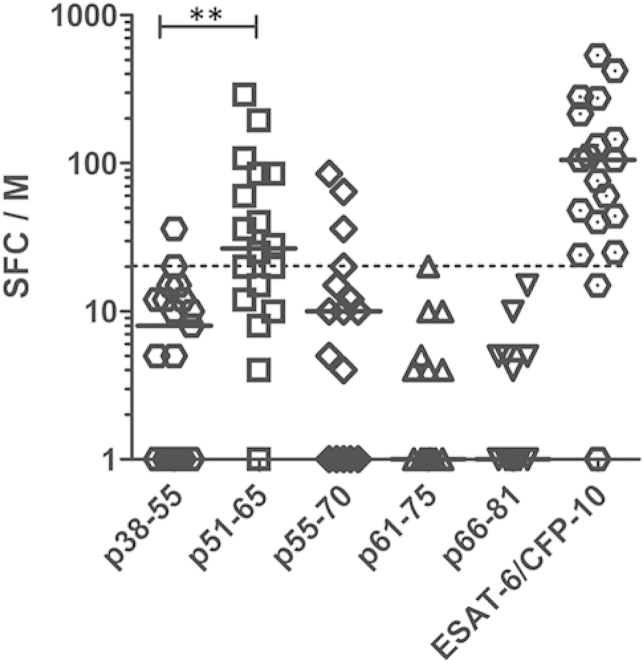
**Detailed analysis of the *C*-terminal 51–81 region of Rv2654c compared to p38-55 peptide**. The number of spot forming cells per million PBMC (SFC/10^6^) are presented from *n* = 19 MTB sensitized individuals. The cut-off for positive response was 20 SFC/10^6^ above nil (dotted line). Significance was calculated using Mann–Whitney U test. **p < 0.005. Black lines represent the median SFC/10^6^ values on both panels.

For the detailed *C*-terminal analysis, 15-mer peptides were prepared (Table 1), in order to fine map the 51–81 amino acid region. As control, the p38-55 peptide was also prepared as this peptide is included in the QuantiFERON-TB Gold In-Tube test (coded as TB7.7 p4). In the combined group of 19 MTB sensitized individuals (representing a subset of the previously studied *n* = 10 active TB patients, and *n* = 9 LTBI, selected based on availability of frozen PBMC remaining after the first set of experiments), the highest IFN-γ response was seen to p51-65, with a median of 27 spot forming cells/million PBMC (SFC/10^6^, IQR 12–85), recognized by 67% of all PBMC samples (Fig. 2). This was significantly higher than the response to the previously described p38-55 peptide (median 8 SFC/10^6^, IQR 0–12; *p*_*corr*_ = 0.002, Mann–Whitney U test with Bonferroni correction), with only 11% of PBMC samples giving a positive response. These results suggest that the most frequently recognized epitope of Rv2654c protein in this population is present within the 51–65 amino acid region.

### Predicted epitope promiscuity in different test populations

**Figure 3:**
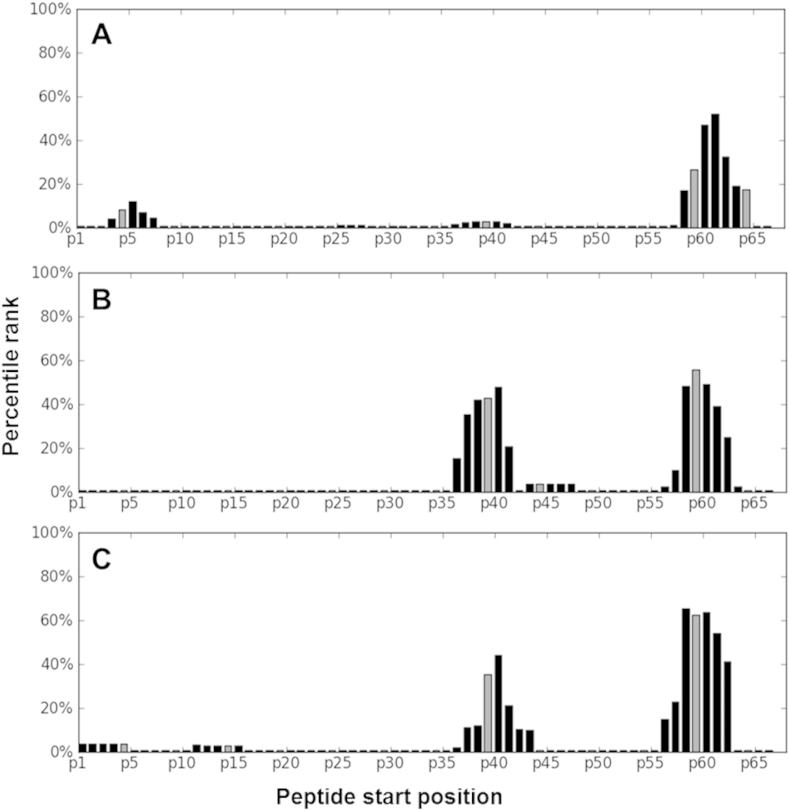
**Population tailored epitope prediction**. The portion of the population able to present a 15-mer peptide was estimated based on simulations combining MHC binding scores of the IEDB epitope prediction tool and HLA serotype frequencies described in the Xhosa population. The 5 best scoring peptides for a given combination of HLA alleles were regarded as *binders* (binding to an APC), with a fair chance for antigen presentation. The number of people likely to have a given combination of HLA alleles was estimated using the Hardy–Weinberg equation. Percentage of the whole population, expected to present a given peptide is given for each possible starting position of 15-mers derived from the Rv2654 protein. Panel (A) represents the percentage of the Xhosa population, expected to present 15-mer peptides, based on both HLA-DR and HLA-DQ predictions of the IEDB tool. On panel (B), percentage of Xhosa population based only on HLA-DR prediction is presented, while on panel (C), percentage of the Danish population, based on HLA-DR predictions is represented.

Since the method suggested by Sinu and colleagues does not take differences in HLA frequencies in different populations into account, we related epitope predictions and our *in vitro* experimental findings to the population tested. In order to achieve this, MHC binding scores of the IEDB epitope prediction tool were corrected for the serotype frequencies of the Xhosa population following a stringent model. We found that only the region near the *C*-terminal end of the protein was immunogenic enough to show up in the analysis (Fig. 3A). If binding only to HLA-DR region was calculated, however, two highly immunogenic regions (starting at positions around 40 and around 60) could be detected (Fig. 3B). The location of these regions was similar in case of both the Xhosa and the Danish populations (Fig. 3C).

### Boosting QuantiFERON-TB gold in-tube test with peptide p51-65

We next evaluated the boosting effect of peptide p51-65 on the QuantiFERON-TB Gold In-Tube (QFT) test in an HIV uninfected (*n* = 55) and an HIV infected (*n* = 44) group of patients, none of whom had any signs and/or symptoms of active TB. The median age of the HIV uninfected participants was 26 (30 female, 25 male) and 33 for the HIV infected (30 female, 14 male) patients. The median CD4^+^ T-cell count in the HIV uninfected cohort was 817 and 197 in the HIV infected group.

In the HIV uninfected group, the quantitative performance of the QFT test increased significantly from a median of 1.83 IU/ml (background – nil, IQR 0.30–7.35) to 2.83 (IQR 0.28–12.2), *p* = 0.002, Table 3. There was a statistically non-significant change in the percentage of responders from 73% to 75%, and in sensitivity from 95% to 98%, due to the peptide boost.

In the case of the HIV infected cohort, the quantitative performance of the QFT and modified QFTB did not differ at day 0 of ART, with medians of 1.17 (IQR 0.26–7.14) and 1.17 IU/ml (IQR 0.05–6.11) respectively (Table 4). However, 3 months into antiretroviral treatment (ART) the frequency of positive responders increased from 57% to 64% (statistically not significant) with 5 patients changing from negative (QFT) to positive (modified QFTB) response. Of these patients, 4 remained positive at 6 months of ART (one was not tested at 6 months of ART, Supplementary Table 2). Additionally, at 6 months another 3 patients changed from negative to positive response due to the peptide boost. Overall, at 6 months of ART, the frequency of positive responders increased from 56% to 64% due to the peptide boost, however, this increase was not statistically significant. Additionally, we calculated sensitivities as a ratio of the number of true positive responders over the number of false negative plus true positive responders. False negativity is defined for those who had at least one positive result before. We found an increased albeit not significant, sensitivity (90% from 80% to 93%–81% at 3 and 6 months, respectively) for QFTB compared to QFT.

## Discussion

Peptide epitopes, which are minimal sequences of proteins necessary for immune recognition, have attracted considerable attention as diagnostic reagents. Peptides are chemically well-defined molecular entities that can be easily synthesized in industrial scale and offer improved purity and specificity. Furthermore, synthetic peptides are generally more stable than full-length proteins or whole organisms, and they do not require refrigerated storage.

Therefore, epitope mapping of the Rv2654c protein was evaluated with the aim to identify antigenic peptides that can be used to induce a cytokine response in an IGRA-type test. Our data indicate that more frequently recognised peptides of Rv2654c in South Africa are in the *C*-terminal region between amino acids 51–81. Further detailed epitope mapping resulted in the identification of peptide p51-65 which was the most dominantly recognised peptide of the Rv2654c protein. It is interesting that when testing the 20-mer peptides, the spot counts were higher for p61-81 as compared to p51-70, which contains the p51-65 sequence. However, the % responders overall were high for both peptides. The strength of peptide binding is influenced by the flanking regions of the epitope and it is possible that the p51-65 sequence allows stronger binding of the epitope region as compared to p51-70. It is also likely that the shared sequence between p61-81 and p51-65, amino acids 61–65 are part of the epitope core, the binding of which is more efficient in the context of the p51-65, in stimulating T cell responses.

This finding contrasts with publications^18^, ^19^ that identified p38-55 as the most potent antigenic peptide. Our observation might be explained by different experimental conditions (overnight ELISpot assay compared to a 5 day proliferation assay combined with the measurement of IFN-γ in the supernatant by ELISA), or by the difference in the HLA frequencies of the South African Xhosa population, where our experiments were performed. Computational prediction suggested that the middle region between amino acids 30–50 of the Rv2654c protein to be unrecognised by the Xhosa population and only the region near the C-terminal section of the protein was predicted to be immunogenic enough to show up in the analysis. These results, together with the results of Sette and his co-workers,^36^ who have also found discrepancy between their study and earlier works, highlight the necessity of taking population dependent HLA restriction in antigen recognition into account.

In terms of chemical considerations, p51-65 bears further advantages compared to p38-55, such as being more soluble and more stable under *in vitro* conditions. These characteristics make peptide p51-65 that provoked significantly increased IFN-γ response in a whole blood assay, a more convenient synthetic antigen.

Using synthetic peptide p51-65 in the QuantiFERON-TB Gold In-Tube assay resulted in significant boosting of the quantitative performance of the QFT test in the HIV uninfected group. This increase in the IFN-γ response is most likely due to the presence of the additional epitope recognised by antigen specific T cells, secreting IFN-γ. As the IFN-γ is measured in the supernatant, the response is additive, hence the increase. In the HIV infected cohort the quantitative performance did not change at day 0 and 1 month into the initiation of antiretroviral treatment. However, 3 months of ART resulted in an enhanced proportion of persons scoring positive in the boosted QFT assay, most likely due to ART induced immune recovery and increased CD4^+^ T-cell numbers, able to recognise additional epitopes. Although the increased recognition was not statistically (and thus also not clinically) significant, is in accordance with the findings of Kellar and colleagues, who have demonstrated that the combination of ESAT-6 and CFP-10 peptide pools with the pool of overlapping peptides representing Rv2654c protein, has resulted in a significantly greater cytokine and chemokine response in TB patients using whole-blood assay.^37^ Moreover, the reason for not seeing a significant increase in median IFN-γ responses in the HIV infected individuals could be due to the relatively short duration of follow up (6 months) on ART. In our previous study^38^ we demonstrated that ART is associated with an absolute increase in effector function (which is what is being measured by IGRA), but the proportional response decreased over 1 year of ART, and the strongest correlate of increased ART-mediated immunity was the central memory response. Thus, in the expanding CD4 T cell pool, containing antigen specific T cells that are potentially able to recognise the additional antigen, these cells are not high enough in numbers to reflect an increase in IFN-γ responses at 6 months of ART.

Overall, different ethnic populations may respond to different peptides than what is available in the commercial IGRA, but the overall effect is a modest increase in responders and not likely to change current assay procedures. While our data are preliminary and warrant further validation with different epitopes, we can conclude that it shows promise for population tailored detection of MTB sensitization and for promiscuous synthetic epitope peptides of proteins such as Rv2654c to be considered as part of more effective immunodiagnostics.

## Conflict of interest

The authors declare no commercial or financial conflict of interest.

## Figures and Tables

**Table 1:** Sequence and characteristics of peptides used in this study.

Code	Sequence	M_av_ calc.	M_av_^a^ meas.	R_t_^b^ [min]
p1-20	MSGHALAARTLLAAADELVG	1966.3	1966.1	31.7
p11-30	LLAAADELVGGPPVEASAAA	1821.1	1821.0	25.8
p21-40	GPPVEASAAALAGDAAGAWR	1837.0	1837.0	27.5
p31-50	LAGDAAGAWRTAAVELARAL	1982.3	1982.1	34.4
p41-60	TAAVELARALVRAVAESHGV	2019.3	2019.2	33.3
p51-70	VRAVAESHGVAAVLFAATAA	1910.2	1909.8	32.0
p61-81	AAVLFAATAAAAAAVDRGDPP	1925.2	1925.1	30.5
p38-55	AWRTAAVELARALVRAVA	1922.1	1922.2	41.5^c^
p51-65	VRAVAESHGVAAVLF	1523.8	1523.8	28.4
p55-70	AESHGVAAVLFAATAA	1483.8	1483.8	30.0
p61-75	AAVLFAATAAAAAAV	1286.7	1286.8	16.5^d^
p66-81	AATAAAAAAVDRGDPP	1422.7	1422.8	16.6^d^

a Average molecular mass measured by a Bruker Esquire 3000 + electrospray mass spectrometer.

b Retention time on an analytical RP-HPLC using an Eurospher-100, C-18, 5 μm, 250 × 4 mm column; gradient: 5% B, 5 min; 5–60% B, 35 min.

c Gradient: 5% B, 5 min; 5–90% B, 45 min.

d Phenomenex Jupiter C-4, 5 μm, 250 × 4 mm column, gradient: 5% B, 5 min; 5–70% B, 35 min.

**Table 2:** Recognition of single peptides and Rv2654c peptide pool by individuals with LTBI and active TB.

	LTBI	*n* = 24		Active	*n* = 10		Combined	*n* = 34	
	No. pos. resp.^a^	Sensitivity % (95% CI)^b^	Median SFC/10^6^ (IQR)^c^	No. pos. resp.^a^	Sensitivity % (95% CI)^b^	Median SFC/10^6^ (IQR)^c^	No. pos. resp.^a^	Sensitivity % (95% CI)^b^	Median SFC/10^6^ (IQR)^c^
p1-20	6/24	25 (9.8–46.7)	5 (0–19)	2/10	20 (2.5–55.6)	3 (0–15)	8/34	24 (10.8–41.2)	4 (0–17)
p11-30	1/24	4 (0.1–21.1)	0 (0–8)	1/10	10 (0.3–44.5)	2 (0–6)	2/34	6 (0.7–19.7)	0 (0–8)
p21-40	4/24	17 (4.7–37.4)	4 (0–10)	2/10	20 (2.5–55.6)	9 (0–28)	6/34	18 (6.8–34.5)	4 (0–11)
p31-50	6/24	25 (9.8–46.7)	6 (0–18)	2/10	20 (2.5–55.6)	9^5^, ^6^, ^7^, ^8^, ^9^, ^10^, ^11^, ^12^, ^13^, ^14^	8/34	24 (10.8–41.2)	7 (0–14)
p41-60	4/24	17 (4.7–37.4)	4 (0–8)	1/10	10 (0.3–44.5)	2 (0–9)	5/34	15 (5.0–31.1)	4 (0–8)
p51-70	7/24	29 (12.6–51.1)	4 (0–23)	3/10	30 (6.7–65.3)	9 (0–26)	10/34	29 (15.1–47.5)	4 (0–21)
p61-81	7/24	29 (12.6–51.1)	8 (0–20)	5/10	50 (18.7–81.3)	18 (5–46)	12/34	35 (19.8–53.5)	10 (0–24)
Rv2654c pool	7/24	29 (12.6–51.1)	6 (0–35)	4/10	40 (12.2–73.8)	10^6^, ^7^, ^8^, ^9^, ^10^, ^11^, ^12^, ^13^, ^14^, ^15^, ^16^, ^17^, ^18^, ^19^, ^20^, ^21^, ^22^, ^23^, ^24^	11/34	32 (17.4–50.5)	8 (0–24)
ESAT-6/CFP-10	24/24	100 (85.8–100)	240 (82–419)	8/10	80 (44.4–97.5)	109 (37–163)	32/34	94 (80.3–99.3)	156 (61–362)

PBMC of either LTBI (*n* = 24) or active TB patients (*n* = 10) were *in vitro* stimulated with 20-mer overlapping peptides spanning the entire sequence of Rv2654c protein using the ELISpot assay. The number of spot forming cells per million PBMC (SFC/10^6^) are presented and compared to ESAT-6/CFP-10 response. The cut-off for positive response was 20 SFC/10^6^.

a Number of positive responders/number tested.

b Sensitivity was calculated as a ratio of the number of true positive responders over the number of false negative plus true positive responders.

c Median spot number and interquartile range.

**Table 3:** Recognition and boosting effect of p51-65 in the HIV negative cohort.

	QFT performance	QFTB performance
Number tested *n* =	55	55
Median IU/ml (IQR)^a^	1.83 (0.32–6.90)	2.83 (0.32–11.54)
Comparison of median IU/ml response^b^	** (*p* = 0.002)	
No. of positive responders^c^	40	41
Responders %^d^	73	75
Sensitivity % (95% CI)^e^	95 (83.8–99.4)	98 (87.1–99.9)

Blood samples of 55 HIV uninfected participant were assayed in commercially available QFT and p51-65 peptide boosted QFT (QFTB). The cut-off for positive recognition was 0.35 IU/ml above nil.

a Median IU/ml value in QFT or QFTB assay and interquartile range.

b Significance was calculated using Wilcoxon matched pairs test. **p < 0.01.

c Number of positive responders/number tested.

d Percentage of responders out of all donors tested.

e Sensitivity was calculated as a ratio of the number of true positive responders over the number of false negative plus true positive responders. False negativity was confirmed by ELISpot using ESAT-6/CFP-10 antigens. The difference in sensitivity statistically is not significant.

**Table 4:** Recognition and boosting effect of p51-65 in the HIV infected cohort.

	Day 0 *n* = 441	1 Month *n* = 41	3 Months *n* = 42	6 Months *n* = 39
*QFT performance*				
Median IU/ml (IQR)^a^	1.17 (0.26–7.14)	1.74 (0.09–11.63)	0.57 (0.07–6.89)	1.20 (0.10–4.53)
No. pos. resp.^b^	30	28	24	22
Responders %^c^	68	68	57	56
Sensitivity % (95% CI)^d^	97 (83.3–99.9)	97 (82.2–99.9)	80 (61.4–92.3)	81 (61.9–93.7)
*QFTB performance*				
Median IU/ml (IQR)^a^	1.17 (0.05–6.11)	1.16 (0.09–17.90)	0.70 (0.15–5.78)	1.19 (0.14–4.46)
No. pos. resp.^b^	28/44	27	27	25
Responders %^c^	64	66	64	64
Sensitivity % (95% CI)^d^	90 (74.3–98.0)	93 (77.2–99.2)	90 (73.5–97.9)	93 (75.7–99.1)
Viral load Median (IQR)	71,291 (38,855–2,27,729)	316 (114–593)	39 (39–77)	39 (39–39)
CD4^+^ T cell number Median (IQR)	197 (121–238)	278 (185–340)	298 (189–363)	307 (247–399)

HIV infected participants were sampled before the initiation of ART and 1, 3 and 6 months after the treatment. Blood samples were assayed in commercially available QFT and p51-65 peptide boosted QFT (QFTB). The cut-off for positive recognition was 0.35 IU/ml above nil.

a Median IU/ml value in QFT or QFTB assay and interquartile range.

b Number of positive responders/number tested.

c Percentage of responders out of all donors tested.

d Sensitivity was calculated as a ratio of the number of true positive responders over the number of false negative plus true positive responders. False negativity was defined for those who had at least one positive result before. There was a statistically non-significant trend of increasing sensitivity at 3 and 6 months for QFTB compared to QFT.

## References

[1] WHO (2014). Global tuberculosis report 2014. http://www.who.int/tb/publications/global_report/en/.

[2] Kaufmann S.H. (2010). Future vaccination strategies against tuberculosis: thinking outside the box. Immunity.

[3] Andersen P., Doherty T.M. (2005). The success and failure of BCG—implications for a novel tuberculosis vaccine. Nat Rev Microbiol.

[4] Farhat M., Greenaway C., Pai M., Menzies D. (2006). False-positive tuberculin skin tests: what is the absolute effect of BCG and non-tuberculous mycobacteria?. Int J Tuberc Lung D.

[5] Garnier T., Eiglmeier K., Camus J.C., Medina N., Mansoor H., Pryor M. (2003). The complete genome sequence of *Mycobacterium bovis*. Proc Natl Acad Sci U. S. A.

[6] Cole S.T., Brosch R., Parkhill J., Garnier T., Churcher C., Harris D. (1998). Deciphering the biology of *Mycobacterium tuberculosis* from the complete genome sequence. Nature.

[7] Mahairas G.G., Sabo P.J., Hickey M.J., Singh D.C., Stover C.K. (1996). Molecular analysis of genetic differences between *Mycobacterium bovis* BCG and virulent *M-bovis*. J Bacteriol.

[8] Behr M.A., Wilson M.A., Gill W.P., Salamon H., Schoolnik G.K., Rane S. (1999). Comparative genomics of BCG vaccines by whole-genome DNA microarray. Science.

[9] Pai M., Denkinger C.M., Kik S.V., Rangaka M.X., Zwerling A., Oxlade O. (2014). Gamma interferon release assays for detection of *Mycobacterium tuberculosis* infection. Clin Microbiol Rev.

[10] Renshaw P.S., Panagiotidou P., Whelan A., Gordon S.V., Hewinson R.G., Williamson R.A. (2002). Conclusive evidence that the major T-cell antigens of the *Mycobacterium tuberculosis* complex ESAT-6 and CFP-10 form a tight, 1:1 complex and characterization of the structural properties of ESAT-6, CFP-10, and the ESAT-6*CFP-10 complex. Implications for pathogenesis and virulence. J Biol Chem.

[11] Guinn K.M., Hickey M.J., Mathur S.K., Zakel K.L., Grotzke J.E., Lewinsohn D.M. (2004). Individual RD1-region genes are required for export of ESAT-6/CFP-10 and for virulence of *Mycobacterium tuberculosis*. Mol Microbiol.

[12] van Ingen J., de Zwaan R., Dekhuijzen R., Boeree M., van Soolingen D. (2009). Region of difference 1 in nontuberculous Mycobacterium species adds a phylogenetic and taxonomical character. J Bacteriol.

[13] Harboe M., Oettinger T., Wiker H.G., Rosenkrands I., Andersen P. (1996). Evidence for occurrence of the ESAT-6 protein in *Mycobacterium tuberculosis* and virulent *Mycobacterium bovis* and for its absence in *Mycobacterium bovis* BCG. Infect Immun.

[14] Esmail H., Barry C.E., Wilkinson R.J. (2012). Understanding latent tuberculosis: the key to improved diagnostic and novel treatment strategies. Drug Discov Today.

[15] Barry C.E., Boshoff H.I., Dartois V., Dick T., Ehrt S., Flynn J. (2009). The spectrum of latent tuberculosis: rethinking the biology and intervention strategies. Nat Rev Microbiol.

[16] Gideon H.P., Wilkinson K.A., Rustad T.R., Oni T., Guio H., Kozak R.A. (2010). Hypoxia induces an immunodominant target of tuberculosis specific T cells absent from common BCG vaccines. PLoS Pathog.

[17] Gideon H.P., Wilkinson K.A., Rustad T.R., Oni T., Guio H., Sherman D.R. (2012). Bioinformatic and empirical analysis of novel hypoxia-inducible targets of the human antituberculosis T cell response. J Immunol.

[18] Aagaard C., Brock I., Olsen A., Ottenhoff T.H., Weldingh K., Andersen P. (2004). Mapping immune reactivity toward Rv2653 and Rv2654: two novel low-molecular-mass antigens found specifically in the *Mycobacterium tuberculosis* complex. J Infect Dis.

[19] Brock I., Weldingh K., Leyten E.M., Arend S.M., Ravn P., Andersen P. (2004). Specific T-cell epitopes for immunoassay-based diagnosis of *Mycobacterium tuberculosis* infection. J Clin Microbiol.

[20] Lew J.M., Kapopoulou A., Jones L.M., Cole S.T. (2011). TubercuList-10 years after. Tuberculosis.

[21] Lew J.M., Mao C., Shukla M., Warren A., Will R., Kuznetsov D. (2013). Database resources for the tuberculosis community. Tuberculosis.

[22] Wang P., Sidney J., Dow C., Mothe B., Sette A., Peters B. (2008). A systematic assessment of MHC class II peptide binding predictions and evaluation of a consensus approach. PLoS Comput Biol.

[23] Wang P., Sidney J., Kim Y., Sette A., Lund O., Nielsen M. (2010). Peptide binding predictions for HLA DR, DP and DQ molecules. BMC Bioinforma.

[24] Vita R., Overton J.A., Greenbaum J.A., Ponomarenko J., Clark J.D., Cantrell J.R. (2015). The immune epitope database (IEDB) 3.0. Nucleic Acids Res.

[25] Greenbaum J., Sidney J., Chung J., Brander C., Peters B., Sette A. (2011). Functional classification of class II human leukocyte antigen (HLA) molecules reveals seven different supertypes and a surprising degree of repertoire sharing across supertypes. Immunogenetics.

[26] Paul S., Lindestam Arlehamn C.S., Scriba T.J., Dillon M.B., Oseroff C., Hinz D. (2015). Development and validation of a broad scheme for prediction of HLA class II restricted T cell epitopes. J Immunol Methods.

[27] Gideon H.P., Phuah J., Myers A.J., Bryson B.D., Rodgers M.A., Coleman M.T. (2015). Variability in tuberculosis granuloma T cell responses exists, but a balance of pro- and anti-inflammatory cytokines is associated with sterilization. PLoS Pathog.

[28] Gideon H.P., Hamilton M.S., Wood K., Pepper D., Oni T., Seldon R. (2013). Impairment of IFN-gamma response to synthetic peptides of *Mycobacterium tuberculosis* in a 7-day whole blood assay. PloS one.

[29] du Toit ED (1988). MacGregor KJ, Taljaard DG, Oudshoorn M. HLA-A, B, C, DR and DQ polymorphisms in three South African population groups: South African Negroes, Cape Coloureds and South African Caucasoids. Tissue Antigens.

[30] Mack S.J., Gourraud P.A., Single R.M., Thomson G., Hollenbach J.A. (2012). Analytical methods for immunogenetic population data. Methods Mol Biol.

[31] Hunter J.D. (2007). Matplotlib: a 2D graphics environment. Comput Sci Eng.

[32] Lindblom B., Svejgaard A., Tsuji K., Aizawa M., Sasazuki T. (1992). HLA genes and haplotypes in the Scandinavian populations. Eleventh International Histocompatibility Workshop and Conference.

[33] Gonzalez-Galarza F.F., Takeshita L.Y., Santos E.J., Kempson F., Maia M.H., da Silva A.L. (2015). Teles e Silva AL, Ghattaoraya GS, Alfirevic A, Jones AR, Middleton D. Allele frequency net 2015 update: new features for HLA epitopes, KIR and disease and HLA adverse drug reaction associations. Nucleic Acids Res.

[34] Arend S.M., Geluk A., van Meijgaarden K.E., van Dissel J.T., Theisen M., Andersen P. (2000). Antigenic equivalence of human T-cell responses to *Mycobacterium tuberculosis-*specific RD1-encoded protein antigens ESAT-6 and culture filtrate protein 10 and to mixtures of synthetic peptides. Infect Immun.

[35] Skjot R.L., Brock I., Arend S.M., Munk M.E., Theisen M., Ottenhoff T.H. (2002). Epitope mapping of the immunodominant antigen TB10.4 and the two homologous proteins TB10.3 and TB12.9, which constitute a subfamily of the esat-6 gene family. Infect Immun.

[36] Arlehamn C.S., Sidney J., Henderson R., Greenbaum J.A., James E.A., Moutaftsi M. (2012). Dissecting mechanisms of immunodominance to the common tuberculosis antigens ESAT-6, CFP10, Rv2031c (hspX), Rv2654c (TB7.7), and Rv1038c (EsxJ). J Immunol.

[37] Kellar K.L., Gehrke J., Weis S.E., Mahmutovic-Mayhew A., Davila B., Zajdowicz M.J. (2011). Multiple cytokines are released when blood from patients with tuberculosis is stimulated with *Mycobacterium tuberculosi*s antigens. PLoS One.

[38] Wilkinson K.A., Seldon R., Meintjes G., Rangaka M.X., Hanekom W.A., Maartens G. (2009). Dissection of regenerating T-Cell responses against tuberculosis in HIV-infected adults sensitized by *Mycobacterium tuberculosis*. Am J Respir Crit Care Med.

