# Supplementary material for: Population tailored modification of tuberculosis specific interferon-gamma release assay
Source: J Infect. 2016 Feb;72(2):179–88. doi: 10.1016/j.jinf.2015.10.012 (PMC4747975; doi:10.1016/j.jinf.2015.10.012)
Supplement: Supplementary file 1 [file mmc1.docx]

**Population tailored modification of tuberculosis specific interferon-gamma release assay**

**Supplementary information:**

**Detailed Description of Epitope Predictions for HLA Frequencies Observed in the Xhosa Population**

To estimate the portion of the Xhosa population expected to present the given epitope, we used a model, where an individual's antigen presenting cells (APCs) express both maternal and paternal HLA-DR and HLA-DQ genes. In case of HLA-DR, an additional pair of HLA-DR3 or HLA-DR4 loci was also expected to be expressed if the individual was serotyped as DR4, DR7 or DR9. The relative number of individuals expressing a given combination of four or six HLA loci was computed according to the Hardy-Weinberg equation. Competition between peptide epitopes was simulated according to two different scenarios.

First, as a less stringent condition, the top scoring 5 peptides (based on the *100 - percentile rank* value) were listed for all MHC molecules expressed on the APCs. In the given subpopulation, all of these 10-30 peptides (depending on the actual genotype) were marked as binders to that portion of the population. Results based on this model, however, are not shown in the paper, since the results of improved, more stringent were found to be more realistic.

In this more stringent model, MHC molecules were also expected to compete for epitopes, so out of the previous 10-30 peptides, only the top scoring 5 peptides were marked as *binders*. The sum of all fractions of the population, where the peptide scored as a *binder*, was regarded as the portion of the whole population that is able to present the peptide epitope.

***Supplementary Table 1.***

**Conversion of serotypes observed in the Xhosa population to genotypes**

| **Serotype** | **Allele** |
| --- | --- |
| DR1 | HLA-DRB1*0101 |
| DR2 | HLA-DRB1*1501 |
| DR3 | HLA-DRB1*0301 |
| DR4 | HLA-DRB1*0401 |
| DR4 | HLA-DRB1*0405 |
| DR5 | HLA-DRB1*1101 |
| DR5 | HLA-DRB1*1201 |
| DRw6 | HLA-DRB1*1302 |
| DR7 | HLA-DRB1*0701 |
| DRw8 | HLA-DRB1*0802 |
| DRw9 | HLA-DRB1*0901 |
| DRw10 | HLA-DRB1*1001 |
| DRw52 | HLA-DRB3*0101 |
| DRw52 | HLA-DRB3*0202 |
| DRw53 | HLA-DRB4*0101 |
| DQw1 | HLA-DQA1*0101/HLA-DQB1*0501 |
| DQw1 | HLA-DQA1*0102/HLA-DQB1*0602 |
| DQw2 | HLA-DQA1*0501/HLA-DQB1*0201 |
| DQw3 | HLA-DQA1*0301/HLA-DQB1*0302 |
| DQw3 | HLA-DQA1*0501/HLA-DQB1*0301 |

HLA binding scores were obtained *in silico*, for individual HLA alleles at gene level, while HLA allele frequencies were available as serotype frequencies. The frequency of a given serotype was regarded as the frequency of the most prevalent HLA allele in that serotype. If the prevalence of two alleles were very close, the two most common alleles shared equal portions of the serotype’s frequency. Serotypes are followed by the corresponding genotype in each row.

***Supplementary Table 2.***

**Results of QFT and peptide boosted QFTB tests in the HIV positive cohort.**

|  | **day 0** | | **1 month** | | **3 months** | | **6 months** | |
| --- | --- | --- | --- | --- | --- | --- | --- | --- |
| ***patient no.*** | **QFT** | **QFTB** | **QFT** | **QFTB** | **QFT** | **QFTB** | **QFT** | **QFTB** |
| RECON1 | 0.25 | 0.18 | 0.49 | 0.81 | 0.6 | 0.73 | 0.24 | 0.33 |
| RECON5 | 0.05 | 0.01 | 0 | 0.01 | 0 | 0 | 0 | 0.14 |
| RECON6 | 0.44 | 0.05 | 0.11 | 0 | 0.07 | 0.01 | 0.09 | 6.15 |
| RECON11 | 3.57 | 0.82 | 0.88 | 1.16 | 0.73 | 0.29 | 0.31 | 0.3 |
| RECON12 | 0.75 | 0.02 | 0.03 | 0.06 | 0.04 | 0 | 0.1 | 0 |
| RECON13 | 7.31 | 4.02 | 9.45 | 9.22 | 0.3 | 0.48 | 1.2 | 0.66 |
| RECON14 | 38.2 | 10.8 | 6.3 | 9.1 | 5.12 | 5.67 | 3.3 | 3.11 |
| RECON15 | 0.98 | 2.18 | 1.93 | 1.07 | 0.34 | 1.03 | 1.86 | 1.36 |
| RECON19 | 0.01 | 0 | n.t. | n.t. | 57.7 | 51.2 | 26.4 | 34.1 |
| RECON20 | 1.25 | 0.7 | 0.23 | 0.24 | 0.07 | 0.4 | 0.29 | 0.35 |
| RECON21 | 0.51 | 0.41 | 0.71 | 0.93 | 0.21 | 0.67 | n.t. | n.t. |
| RECON23 | 0.27 | 0.72 | n.t. | n.t. | 0.35 | 0.8 | 0.4 | 0.57 |
| RECON24 | 0.77 | 0.36 | 0.44 | 0 | 0.3 | 0.25 | 0.33 | 0.2 |
| RECON25 | 0.97 | 0.18 | 0.08 | 0.11 | 0.06 | 0.16 | 0.19 | 0.12 |
| RECON26 | 0.13 | 0.03 | 0.09 | 0.12 | 0.05 | 0 | 0.18 | 0.01 |
| RECON29 | 0 | 0 | 0.06 | 0 | 0.54 | 0.04 | 0.13 | 0 |
| RECON31 | 0.26 | 0 | 0.19 | 0.36 | 0.82 | 0.64 | n.t. | n.t. |
| RECON35 | 0.05 | 0.09 | 0.07 | 0.05 | 0.04 | 0 | 0.1 | 0.04 |
| RECON36 | 0.31 | 0 | 0.01 | 0 | 0 | 0.06 | 0.01 | 0 |
| RECON38 | 0.06 | 0 | 307 | 197 | 35.2 | 58.9 | n.t. | n.t. |
| RECON41 | 0.31 | 0.06 | 215 | 611 | 0.18 | 0.21 | 0 | 1.26 |
| RECON43 | 0 | 0.01 | 0.08 | 0.01 | 0.01 | 0.02 | 0 | 0.15 |
| RECON46 | 0 | 0 | 0 | 0 | 0.2 | 0.13 | 0 | 0 |
| RECON47 | 0.08 | 0.18 | 0.06 | 0.21 | 0.18 | 0.19 | 0.04 | 0.01 |
| RECON48 | 0 | 0 | 0.02 | 0 | 0.03 | 0.02 | 0 | 0 |
| RECON49 | 1.17 | 1.27 | 0.95 | 0.98 | 0 | 0.39 | 2.53 | 0.85 |

Numbers represents the IU/ml values for patients who give negative response (< 0.35 IU/mL) at least once during the six months period. Orange background represent the negative response, red background is for QFT positivity, QFTB negativity, while green background is used when the response turned into positive due to the peptide boost. (n.t. means ‘not tested’).
